# Using Machine Learning to Predict the In-Hospital Mortality in Women with ST-Segment Elevation Myocardial Infarction

**DOI:** 10.31083/j.rcm2405126

**Published:** 2023-04-24

**Authors:** Pengyu Zhao, Chang Liu, Chao Zhang, Yonghong Hou, Xiaomeng Zhang, Jia Zhao, Guolei Sun, Jia Zhou

**Affiliations:** ^1^Department of Communication Engineering, School of Electrical and Information Engineering, Tianjin University, 300072 Tianjin, China; ^2^Department of Emergency, Thoracic Clinical College, Tianjin Medical University, 300070 Tianjin, China; ^3^Department of Emergency, Tianjin Jinnan Hospital, 300350 Tianjin, China; ^4^Department of Cardiology, Tianjin Chest Hospital, 300222 Tianjin, China

**Keywords:** in-hospital mortality, machine learning, prediction model, SHAP value, STEMI, women

## Abstract

**Background:**

Several studies have shown that women have a higher mortality rate than do men from ST-segment elevation myocardial infarction (STEMI). The present study was aimed at developing a new risk-prediction model for all-cause in-hospital mortality in women with STEMI, using predictors that can be obtained at the time of initial evaluation.

**Methods:**

We enrolled 8158 patients who were admitted with STEMI to the Tianjin Chest Hospital and divided them into two groups according to hospital outcomes. The patient data were randomly split into a training set (75%) and a testing set (25%), and the training set was preprocessed by adaptive synthetic (ADASYN) sampling. Four commonly used machine-learning (ML) algorithms were selected for the development of models; the models were optimized by 10-fold cross-validation and grid search. The performance of all-population-derived models and female-specific models in predicting in-hospital mortality in women with STEMI was compared by several metrics, including accuracy, specificity, sensitivity, G-mean, and area under the curve (AUC). Finally, the SHapley Additive exPlanations (SHAP) value was applied to explain the models.

**Results:**

The performance of models was significantly improved by ADASYN. In the overall population, the support vector machine (SVM) combined with ADASYN achieved the best performance. However, it performed poorly in women with STEMI. Conversely, the proposed female-specific models performed well in women with STEMI, and the best performing model achieved 72.25% accuracy, 82.14% sensitivity, 71.69% specificity, 76.74% G-mean and 79.26% AUC. The accuracy and G-mean of the female-specific model were greater than the all-population-derived model by 34.64% and 9.07%, respectively.

**Conclusions:**

A machine-learning-based female-specific model can conveniently and effectively identify high-risk female STEMI patients who often suffer from an incorrect or delayed management.

## 1. Introduction

ST-elevation myocardial infarction (STEMI), the most serious type of cardiovascular disease, is one of the leading causes of mortality worldwide [1, 2, 3]. Multiple longitudinal studies have shown that mortality from STEMI is higher in women than in men [4, 5, 6, 7, 8]. Risk stratification is critical in identifying high-risk patients and assisting physicians in decision making [9, 10]. The traditional risk assessment tools are the Global Registry of Acute Coronary Events (GRACE) [11] score and the Thrombolysis in Myocardial Infarction (TIMI) score [12, 13], but the following three conditions are usually taken as major limitations for these tools: (1) the predictors are not immediately available on admission, and medical history is unreliable; (2) these tools were used without accounting for sex-specific disease characteristics of STEMI, whereas growing evidence has demonstrated sex differences in both symptom presentation and management efficacy STEMI patients [8, 14]. The symptoms of myocardial infarction (MI) in women patients are atypical, which make women often suffer from an incorrect or delayed management [15, 16, 17, 18, 19]; (3) These tools were developed based on a traditional statistical method, which may lead to the loss of important information [20, 21, 22, 23]. Recently, the GRACE 3.0 score, based on machine learning (ML), was developed to reduce sex inequalities, but it was specially designed for the risk assessment of non-ST-elevation acute coronary syndromes (NSTE-ACS) [7]. Therefore, it is necessary to develop a new risk-prediction model for women with STEMI using predictors that can be obtained at the time of initial evaluation.

ML algorithms can capture nonlinear relationships among clinical variables, and have many successful applications [24, 25, 26, 27, 28]. However, real-world medical data are often imbalanced. When trained with imbalanced data, the developed ML models can be overwhelmed by the majority class (i.e., survival group) and can ignore the minority class (i.e., death group) [29], which is the focus of clinical attention. To alleviate this problem, an effective strategy is data preprocessing. The data-preprocessing approach is to resample the imbalanced training set prior to model training. In order to create the balanced training set, the original imbalanced data set can be oversampled for the minority class and/or undersampled for the majority class [30]. Since the undersampling strategy leads to the loss of information from the majority class, we adopted the adaptive synthetic (ADASYN) oversampling approach [31], which has been proven effective [32]. Due to the “black box” nature of ML algorithms, the SHapley Additive exPlanations (SHAP) value was employed to explain the predictors’ impact on the outcome [33].

The aims of this study were to: (1) develop prediction models for all-cause in-hospital mortality in women with STEMI using four commonly used ML algorithms combined with the ADASYN sampling approach, and (2) explain the prediction models with SHAP values.

## 2. Materials and Methods

### 2.1 Study Sample

**Fig. 1.**
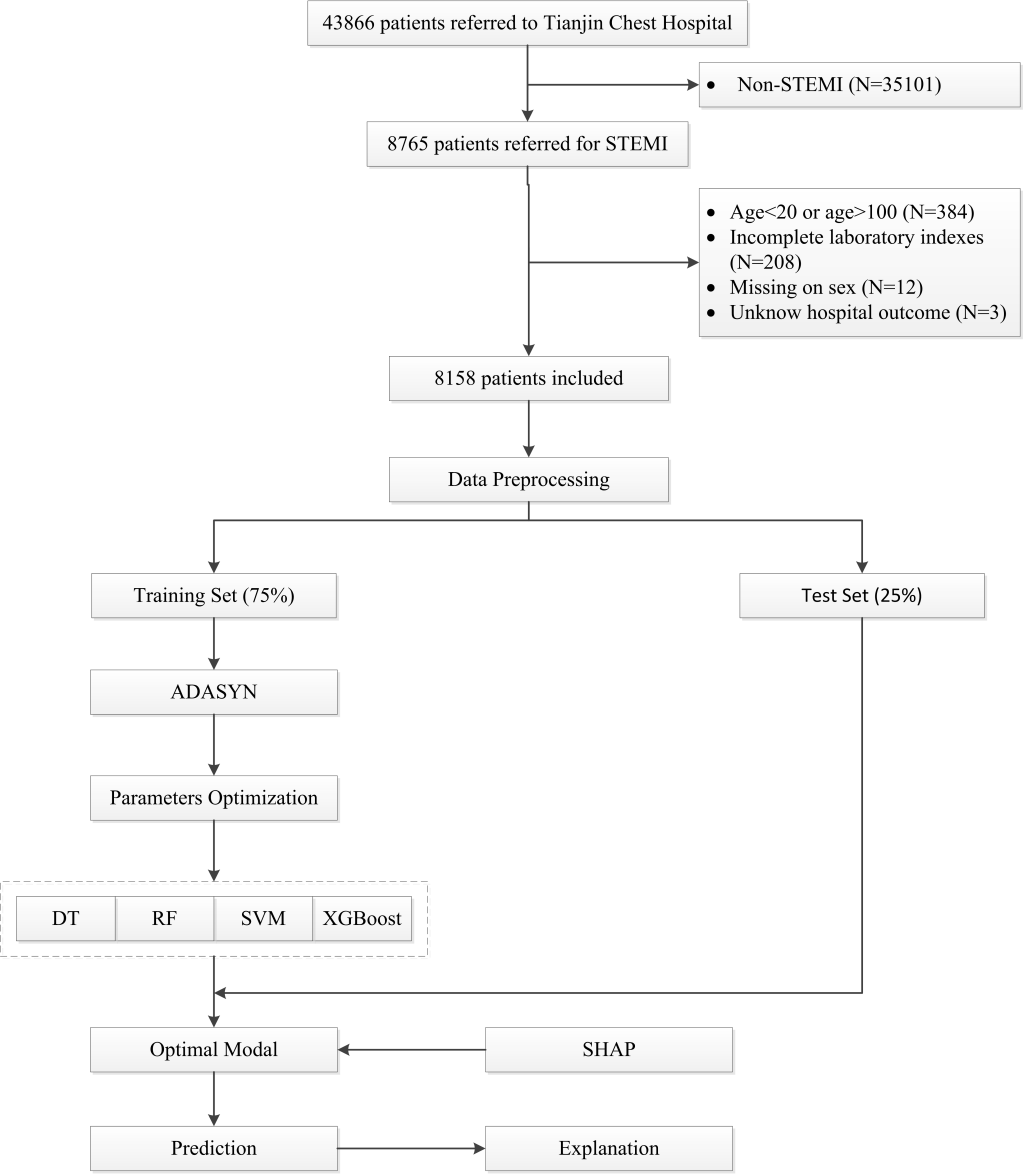
**Flowchart of the study**. STEMI, ST-elevation myocardial infarction; ADASYN, adaptive synthetic; DT, decision trees; RF, random forests; SVM, support vector machines; XGBoost, extreme gradient boosting; SHAP, SHapley Additive exPlanations.

The present study was conducted with information from a hospital-based dataset as described previously [24]. In brief, a total of 8158 patients, from January 2015 to December 2021, with STEMI, were retrospectively enrolled. This sample included 6084 (74.58%) males and 2074 (25.42%) females. The enrollment criteria for patients are as follows: (1) the diagnosis of STEMI complied with the European Society of Cardiology Guidelines for the diagnosis and treatment of acute ST-segment elevation myocardial infarction [34]; (2) persistent ischemic chest pain for less than 12 hours; (3) electrocardiogram (ECG) findings showing the presence of ST segment elevation in two or more consecutive leads, with ≥0.2 mV in the precordial leads and ≥0.1 mV in the limb leads. The exclusion criteria were as follows: (1) age <20 or >100; (2) incomplete laboratory indexes; (3) missing data on sex; or (4) unknown in-hospital outcome. This observational and retrospective study was approved by the Local Ethics Committee. The flowchart of this study is shown in Fig. 1.

### 2.2 Data Collection and Preprocessing

The basic clinical data of the patients were collected, including demographic information (sex, age), physical examination (heart rate, systolic blood pressure, diastolic blood pressure, etc.), laboratory tests (cardiac troponin I), admission pathway, and treatment. All the clinical variables could be obtained at the time of initial evaluation. The primary endpoint was all-cause in-hospital mortality.

Variables with a missing-data percentage of less than 20% were retained. For continuous variables, the mean imputation method was used to supply the missing values, which replaces the missing values of a certain variable with the mean of the available cases. For categorical variables, the mode imputation method was applied to supply the missing values, which replaces the missing values of a certain variable with the mode of the available cases. Respiration, heart rate, systolic blood pressure, diastolic blood pressure, cardiac troponin I, and time from symptom to first medical contact, were missing in 0.06%, 0.05%, 0.07%, 0.07%, 3.36% and 0.33% cases, respectively. Because the range of different variables varied widely, and some of the used algorithms required quantitative data normalization, z-score normalization was used [35].

### 2.3 Statistical Analysis

Categorical variables are reported as counts (%) and continuous variables as mean (SD) or median (IQR). The Kolmogorov-Smirnov test was used to test the normality of distribution. We used Student’s *t* test to assess the differences between parametric continuous variables and the Mann-Whitney-U test for non-parametric variables. We used the Chi-squared test to evaluate the differences between categorical variables. All statistical analysis were performed using Python 3.7.3 (Python Software Foundation, Wilmington, Delaware, USA) with the scientific libraries “scipy.stats”. A two-tailed *p *≤ 0.05 was considered statistically significant.

### 2.4 Model Development and Validation

**Fig. 2.**
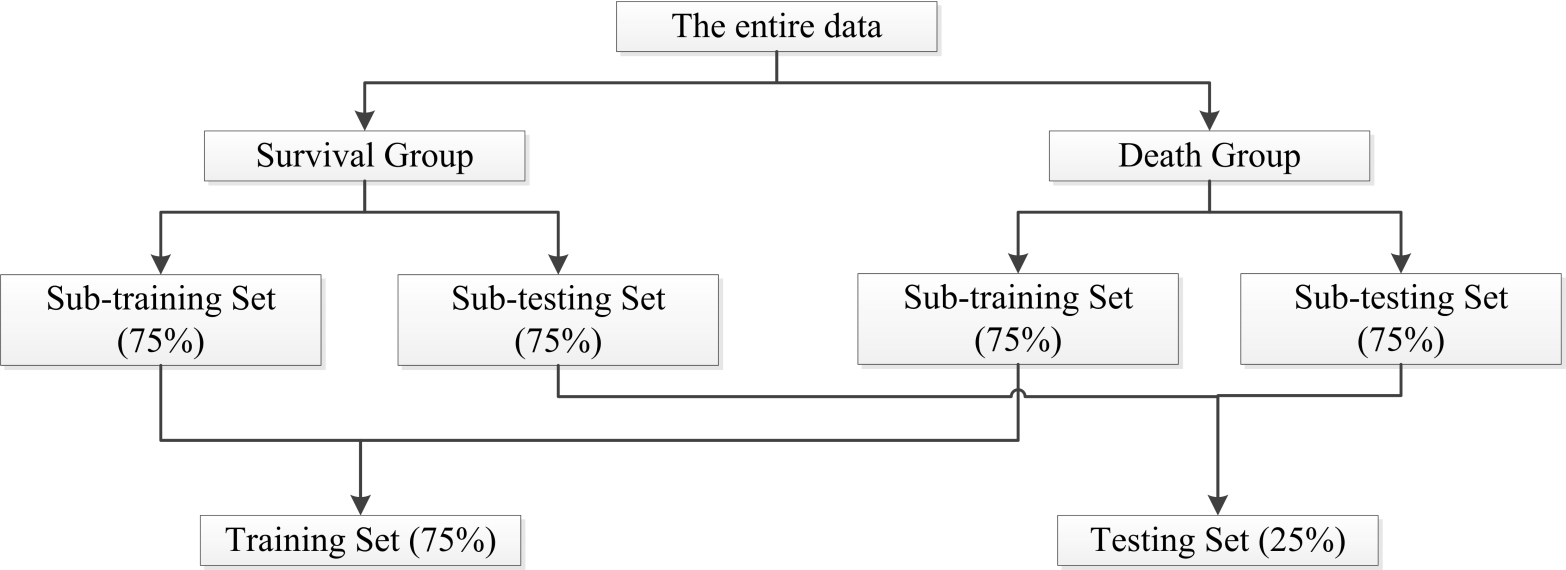
**Flowchart on splitting training and testing sets**.

According to whether the endpoint occurred, the entire set of data was divided into a survival group and a death group. Each of the two groups was randomly split into a sub-training set (75%) and a sub-testing set (25%), and then the two sub-training sets were merged to get the Training Set, and the two sub-testing sets were merged to get the Testing Set as shown in Fig. 2. The Training Set was pretreated using the ADASYN sampling technique to achieve a balance between the minority class (death group) and the majority class (survival group). Four commonly used ML algorithms, including decision trees (DT), random forests (RF), support vector machines (SVM), and extreme gradient boosting (XGBoost), were selected for the development of models to predict the in-hospital mortality in patients with STEMI. A Grid Search method with 10-fold cross validation was used to optimize the ML models. The hyperparameter settings of each model were shown in Table 1. Model performance was assessed according to several learning metrics (accuracy, specificity, sensitivity, G-mean, and area under the receiver operating characteristic curve [AUC]). The performance of all-population-derived models and female-specific models in predicting in-hospital mortality in women with STEMI was compared to demonstrate the effectiveness of the female-specific model proposed in this study. In addition, a 5 × 2 cross validation paired *t* test was used to evaluate the difference between two models [36]. The model development and validation were performed using Python (Version 3.7.3) software with the packages “scikit-learn”, “xgboost”, and “imblearn”.

**Table 1.** **The hyperparameter settings of each model**.

	DT	RF	SVM	XGBoost
‘criterion’	[‘entropy’, ‘gini’]	[‘entropy’, ‘gini’]	-	-
‘max_depth’	range(1, 30)	range(1, 30)	-	range(1, 30)
‘min_samples_split’	range(2, 30)	range(2, 30)	-	-
‘min_samples_leaf’	range(1, 15)	range(1, 15)	-	-
‘n_estimators’	-	range(1, 300)	-	-
‘max_feature’	-	range(2, 12)	-	-
‘kernel’	-	-	[‘linear’, ‘poly’, ‘sigmoid’, ‘rbf’]	-
‘C’	-	-	np.linspace(0.01, 30, 50)	-
‘gamma’	-	-	np.logspace(–10, 1, 50)	np.logspace(–10, 1, 50)
‘coef0’	-	-	np.linspace(0, 5, 10)	-
‘degree’	-	-	[1, 2, 3, 4]	-
‘num_round’	-	-	-	range(1, 300)
‘eta’	-	-	-	np.linspace(0.01, 0.3, 100)
‘sub_sample’	-	-	-	np.linspace(0.1, 1, 10)
‘colsample_bytree’	-	-	-	np.linspace(0.1, 1, 10)
‘colsample_bylevel’	-	-	-	np.linspace(0.1, 1, 10)
‘colsample_bynode’	-	-	-	np.linspace(0.1, 1, 10)
‘lambda’	-	-	-	[0, 1]
‘alpha’	-	-	-	[0, 1]

DT, decision tree; RF, random forest; XGBoost, extreme gradient boosting; SVM, support vector machine.

### 2.5 Model Interpretation

Although the ML models can provide more accurate predictions than traditional statistical models, the results cannot be explained. To show the decision-making process in an intuitive way, the SHAP value was included. SHAP is an approach based on game theory, proposed by Lundberg and Lee, to interpret ML models [33]. The optimal SHAP value was calculated for each feature of each sample after the model was trained, and the impact of each feature on predictions can be represented by SHAP values [37]. Note: the SHAP value has a stronger theoretical basis than other methods [38] and the performance of its explainability has been validated in previous work [25, 39, 40]. The ML model explanation was performed using Python (Version 3.7.3) software with the package “shap”.

## 3. Results

### 3.1 Patient Characteristics

In all, 8158 STEMI patients were included in this study, including 6084 male patients (74.58%) with a median age of 61.00 (53.00, 68.00) years, and 2074 female patients (25.42%) with a median age of 70.00 (63.00, 77.00) years. The median age of all patients was 63.00 (55.00, 71.00) years. The overall in-hospital mortality rate was 3.02% (*n* = 246). Table 2 shows the baseline characteristics and the comparisons between patients who died and those who survived. Compared with surviving patients, dead patients were more likely to have had higher rates of emergency medical services (EMS) admissions, higher Killip classification, lower reperfusion rates, higher age, faster respiration, higher heart rates (HR), lower systolic blood pressure (SBP), lower diastolic blood pressure (DBP) and higher cardiac troponin I (cTnI). Additionally, patients in death group were more likely to have been unconscious.

**Table 2.** **Basic Characteristics of the overall sample by outcome**.

Features		Total	Patients survived	Patients died	*p*-value
No. of patients		8158	7912	246	
Male		6084 (74.58%)	5940 (75.08%)	144 (58.54%)	<0.0001
Consciousness		8135 (99.72%)	7897 (99.81%)	238 (96.75%)	<0.0001
Prehospital mode of transport					<0.0001
	EMS	1406 (17.23%)	1338 (16.91%)	68 (27.64%)	
	Transferred from other hospitals	1475 (18.08%)	1417 (17.91%)	58 (23.58%)	
	Self-transported	5277 (64.69%)	5157 (65.18%)	120 (48.78%)	
Killip classification					<0.0001
	I	7608 (93.26%)	7740 (94.03%)	168 (68.29%)	
	II	405 (4.96%)	366 (4.63%)	39 (15.85%)	
	III	63 (0.77%)	58 (0.73%)	5 (2.03%)	
	IV	82 (1.01%)	48 (0.61%)	34 (13.82%)	
Reperfusion type					<0.0001
	Primary PCI	6028 (73.89%)	5927 (74.91%)	101 (41.06%)	
	Thrombolysis	388 (4.76%)	369 (4.66%)	19 (7.72%)	
	Thrombolysis + Primary PCI	188 (2.30%)	186 (2.35%)	2 (0.81%)	
	Non	1554 (19.05%)	1430 (18.07%)	124 (50.41%)	
Age, years		63.00 (55.00, 71.00)	63.00 (55.00, 71.00)	73.00 (64.00, 80.75)	<0.0001
Respiration, counts/min		18.00 (17.00, 20.00)	18.00 (17.00, 20.00)	19.00 (17.00, 20.00)	0.0277
HR, beats/min		76.00 (65.00, 88.00)	76.00 (65.00, 88.00)	80.00 (69.00, 98.75)	<0.0001
SBP, mm Hg		135.00 (119.00, 151.00)	135.00 (120.00, 151.00)	123.00 (100.25, 140.75)	<0.0001
DBP, mm Hg		80.00 (70.00, 92.00)	81.00 (70.00, 92.25)	76.00 (64.00, 88.00)	<0.0001
cTnI, ng/mL		1.40 (0.12, 3.66)	1.40 (0.12, 3.63)	1.84 (0.27, 4.44)	0.0012
S to FMC, min		118 (62.00, 291.00)	118.00 (62.00, 290.25)	114.00 (62.00, 348.00)	0.6074

EMS, Emergency medical services; PCI, percutaneous coronary intervention; HR, heart rate; SBP, systolic blood pressure; DBP, diastolic blood pressure; cTnI, cardiac troponin I; S to FMC, time from symptom to first medical contact.

### 3.2 Development of All-Population-Derived Models and Validation in Women

**Fig. 3.**
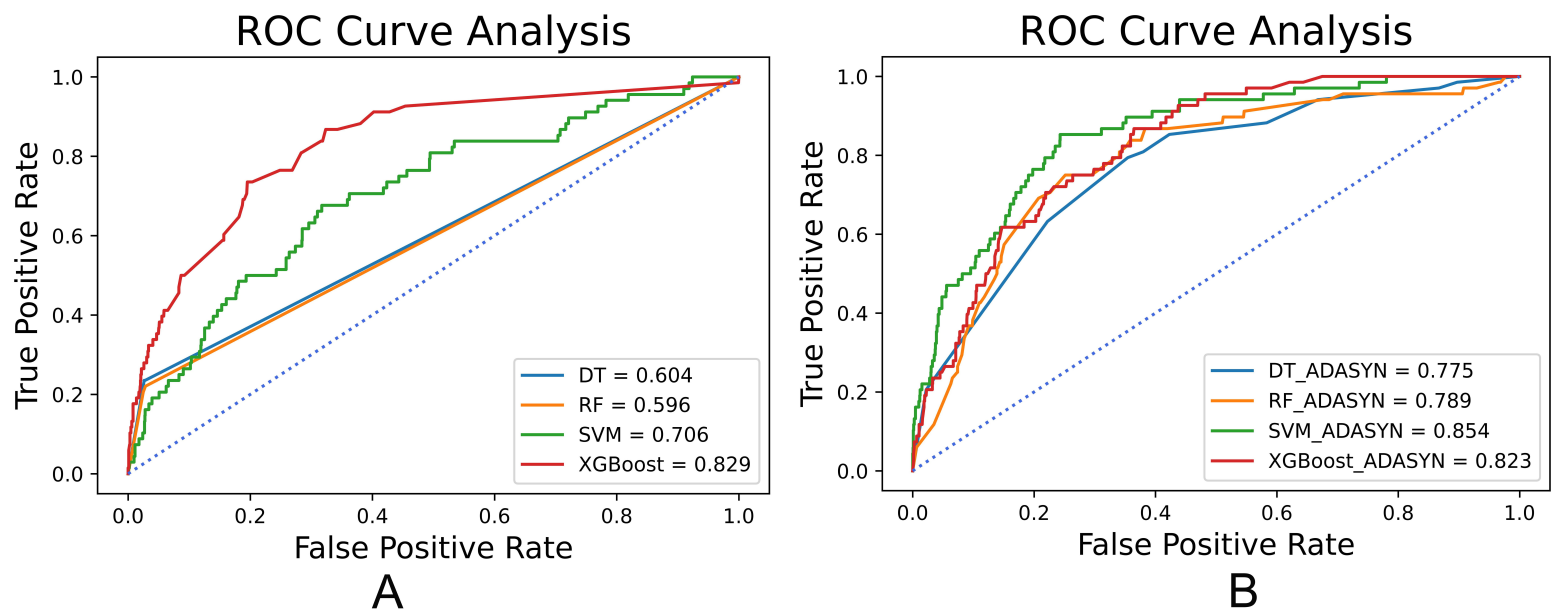
**ROC analysis results of all-population-derived models**. (A) ROC analysis results of models combined without ADASYN. (B) ROC analysis results of models combined with ADASYN. ROC, receiver operating characteristic; ADASYN, adaptive synthetic; DT, decision trees; RF, random forests; SVM, support vector machines; XGBoost, extreme gradient boosting.

**Fig. 4.**
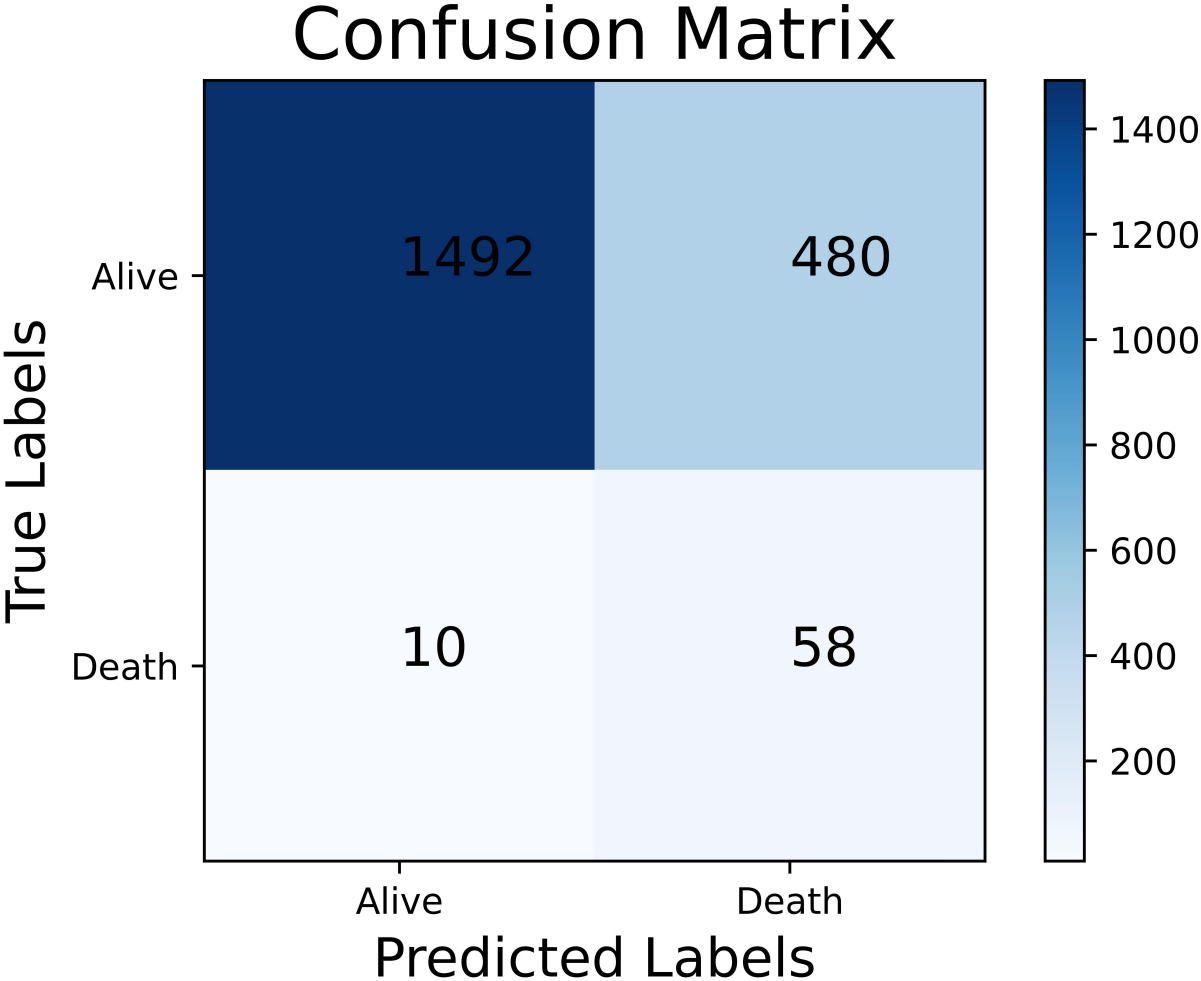
**The confusion matrix of the best performing all-population-derived model in the overall population**.

**Fig. 5.**
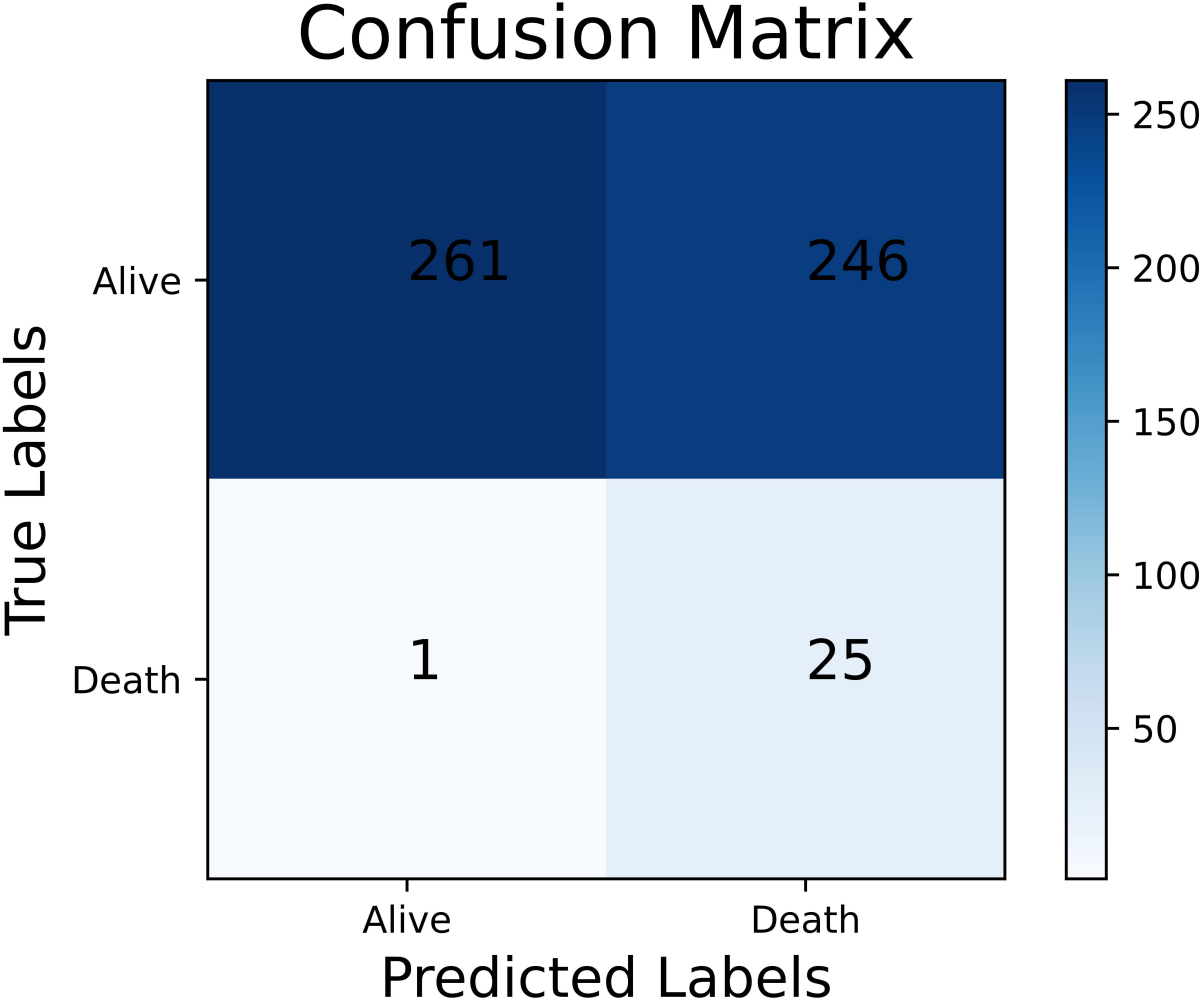
** The confusion matrix of the best performing all-population-derived model in women with STEMI**. STEMI, ST-elevation myocardial infarction.

**Fig. 6.**
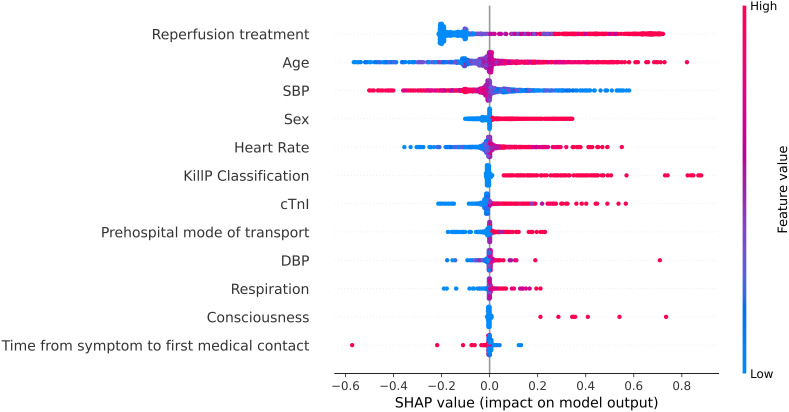
**SHAP values for mortality risk provided by the best performing all-population-derived model**. SBP, systolic blood pressure; cTnI, cardiac troponin I; DBP, diastolic blood pressure; SHAP, SHapley Additive exPlanations.

The performance of different all-population-derived models was shown in Table 3 and the analysis of receiver operating characteristic (ROC) curves was shown in Fig. 3. The performance of models was significantly improved by ADASYN according to G-mean and AUC. The SVM combined with ADASYN achieved the best performance (G-mean: 80.33%; accuracy: 75.98%; sensitivity: 85.29%; specificity: 75.66%; and AUC: 85.36%). As shown in Fig. 4, 1492 of 1972 patients and 58 of 68 patients were correctly classified into the low-risk group and high-risk group, respectively. However, the all-population-derived models performed poorly in women with STEMI as shown in Table 4. The best performing model achieved only 53.66% accuracy, 51.48% specificity and 70.36% G-mean. Fig. 5 shows that 246 of 507 patients were incorrectly classified into the high-risk group. Additionally, Fig. 6 shows that sex (ranked as 4/12) was highly associated with the outcome, and that women have a higher risk of all-cause mortality.

**Table 3.** **The performance of different all-population-derived models in the overall population**.

Model	Accuracy	Specificity	Sensitivity	G-mean	AUC
DT	94.90%	97.36%	23.53%	47.86%	60.39%
DT_ADASYN	65.05%	64.55%	79.41%	71.60%	77.48%
RF	94.95%	97.52%	20.59%	44.81%	59.63%
RF_ADASYN	74.85%	74.85%	75.00%	74.92%	78.86%
SVM	94.07%	96.75%	16.18%	39.56%	70.60%
SVM_ADASYN	75.98%	75.66%	85.29%	80.33%	85.36%
XGBoost	96.47%	99.19%	17.65%	41.84%	82.92%
XGBoost_ADASYN	73.97%	73.99%	73.53%	73.76%	82.33%

DT, decision tree; RF, random forest; SVM, support vector machine; XGBoost, extreme gradient boosting; ADASYN, adaptive synthetic; AUC, area under the curve. G-mean is the geometric mean of sensitivity and specificity.

**Table 4.** **The performance of all-population-derived models in women with STEMI**.

Model	Accuracy	Specificity	Sensitivity	G-mean	AUC
DT	92.87%	96.65%	19.23%	43.11%	57.70%
DT_ADASYN	51.78%	50.49%	76.92%	62.32%	73.27%
RF	92.31%	96.25%	15.38%	38.48%	57.50%
RF_ADASYN	57.60%	56.02%	88.46%	70.39%	75.75%
SVM	92.87%	96.65%	19.23%	43.11%	70.59%
SVM_ADASYN	53.66%	51.48%	96.15%	70.36%	84.71%
XGBoost	95.12%	98.82%	23.08%	47.75%	79.70%
XGBoost_ADASYN	57.97%	56.41%	88.46%	70.64%	79.40%

DT, decision tree; RF, random forest; SVM, support vector machine; XGBoost, extreme gradient boosting; ADASYN, adaptive synthetic; AUC, area under the curve. G-mean is the geometric mean of sensitivity and specificity.

### 3.3 Sex Differences in Patients with STEMI

The comparison between men and women is shown in Table 5. Compared with men, women were more likely to have higher mortality, higher Killip classification, lower reperfusion rates, higher age, lower DBP, and longer time from symptom to first medical contact (S to FMC). The baseline characteristics and the comparisons between the survival group and the death group in female patients were shown in Table 6. Compared with surviving patients, dead patients had been more likely to have higher EMS admission rates, higher Killip classification, lower reperfusion rates, higher age, lower SBP and higher cTnI.

**Table 5.** **Basic Characteristics of the overall patient population by sex**.

Features		Total	Men	Women	*p*-value
No. of patients		8158	6084	2074	
Consciousness		8135 (99.72%)	6069 (99.75%)	2066 (99.61%)	0.4280
Death		246 (3.02%)	144 (2.37%)	102 (4.92%)	<0.0001
Prehospital mode of transport					0.3390
	EMS	1406 (17.23%)	1050 (17.26%)	356 (17.16%)	
	Transferred from other hospitals	1475 (18.08%)	1078 (17.72%)	397 (19.14%)	
	Self-transported	5277 (64.69%)	3956 (65.02%)	1321 (63.69%)	
Killip classification					0.0056
	I	7608 (93.26%)	5708 (93.82%)	1900 (91.61%)	
	II	405 (4.96%)	277 (4.55%)	128 (6.17%)	
	III	63 (0.77%)	41 (0.67%)	22 (1.06%)	
	IV	82 (1.01%)	58 (0.95%)	24 (1.16%)	
Reperfusion type					<0.0001
	Primary PCI	6028 (73.89%)	4581 (75.30%)	1447 (69.77%)	
	Thrombolysis	388 (4.76%)	304 (4.99%)	84 (4.05%)	
	Thrombolysis + Primary PCI	188 (2.30%)	158 (2.60%)	30 (1.45%)	
	Non	1554 (19.05%)	1041 (17.11%)	513 (24.73%)	
Age, years		63.00 (55.00, 71.00)	61.00 (53.00, 68.00)	70.00 (63.00, 77.00)	<0.0001
Respiration, counts/min		18.00 (17.00, 20.00)	18.00 (17.00, 20.00)	18.00 (17.00, 20.00)	0.2218
HR, beats/min		76.00 (65.00, 88.00)	76.00 (65.00, 88.00)	75.00 (64.00, 88.00)	0.0742
SBP, mm Hg		135.00 (119.00, 151.00)	134.00 (120.00, 150.00)	135.00 (119.00, 153.00)	0.3254
DBP, mm Hg		80.00 (70.00, 92.00)	82.00 (71.00, 94.00)	79.00 (70.00, 90.00)	<0.0001
cTnI, ng/mL		1.40 (0.12, 3.66)	1.40 (0.10, 3.69)	1.40 (0.20, 3.58)	0.0870
S to FMC, min		118 (62.00, 291.00)	116.00 (61.00, 281.00)	121.00 (68.00, 327.75)	0.0006

EMS, Emergency medical services; PCI, percutaneous coronary intervention; HR, heart rate; SBP, systolic blood pressures; DBP, diastolic blood pressure; cTnI, cardiac troponin I; S to FMC, time from symptom to first medical contact.

**Table 6.** **Basic Characteristics of female patients by outcome**.

Features		Total	Patients survived	Patients died	*p*-value
No. of patients		2074	1972	102	
Consciousness		2066 (99.61%)	1966 (99.70%)	100 (98.04%)	0.0699
Prehospital mode of transport					<0.0001
	EMS	356 (17.16%)	328 (16.63%)	28 (27.45%)	
	Transferred from other hospitals	397 (19.14%)	370 (18.76%)	27 (26.47%)	
	Self-transported	1321 (63.69%)	1274 (64.60%)	47 (46.08%)	
Killip classification					<0.0001
	I	1900 (91.61%)	1828 (92.70%)	72 (70.59%)	
	II	128 (6.17%)	110 (5.58%)	18 (17.65%)	
	III	22 (1.06%)	21 (1.06%)	1 (0.98%)	
	IV	24 (1.16%)	13 (0.66%)	11 (10.78%)	
Reperfusion type					<0.0001
	Primary PCI	1447 (69.77%)	1406 (71.30%)	41 (40.20%)	
	Thrombolysis	84 (4.05%)	72 (3.65%)	12 (11.76%)	
	Thrombolysis + Primary PCI	30 (1.45%)	30 (1.52%)	0 (0.00%)	
	Non	513 (24.73%)	464 (23.53%)	49 (48.04%)	
Age, years		70.00 (63.00, 77.00)	70.00 (63.00, 77.00)	77.00 (70.25, 83.00)	<0.0001
Respiration, counts/min		18.00 (17.00, 20.00)	18.00 (17.00, 20.00)	19.00 (18.00, 20.00)	0.0545
HR, beats/min		75.00 (64.00, 88.00)	75.00 (64.00, 88.00)	78.00 (65.25, 94.75)	0.0693
SBP, mm Hg		135.00 (119.00, 153.00)	135.00 (119.00, 153.00)	126.50 (101.00, 149.75)	<0.001
DBP, mm Hg		79.00 (70.00, 90.00)	79.00 (70.00, 90.00)	77.00 (65.25, 86.00)	0.0936
cTnI, ng/mL		1.40 (0.20, 3.58)	1.40 (0.18, 3.54)	2.02 (0.62, 4.79)	0.0077
S to FMC, min		121.00 (68.00, 327.75)	121.00 (67.00, 325.00)	125.50 (79.25, 394.75)	0.3927

EMS, Emergency medical services; PCI, percutaneous coronary intervention; HR, heart rate; SBP, systolic blood pressures; DBP, diastolic blood pressure; cTnI, cardiac troponin I; S to FMC, time from symptom to first medical contact.

### 3.4 Development, Validation and Comparison of Female-Specific Models

**Fig. 7.**
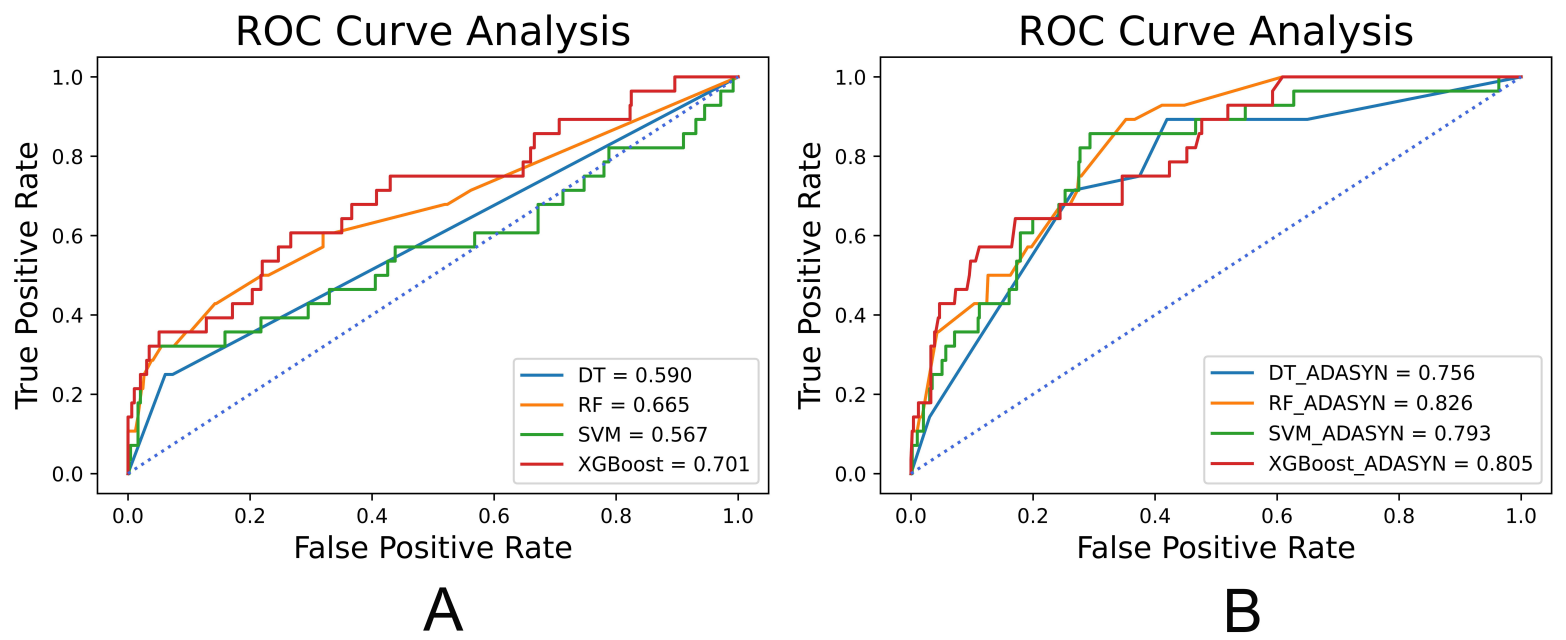
**ROC analysis results of female-specific models**. (A) ROC analysis results of models combined without ADASYN. (B) ROC analysis results of models combined with ADASYN. ROC, receiver operating characteristic; ADASYN, adaptive synthetic; DT, decision trees; RF, random forests; SVM, support vector machines; XGBoost, extreme gradient boosting.

**Fig. 8.**
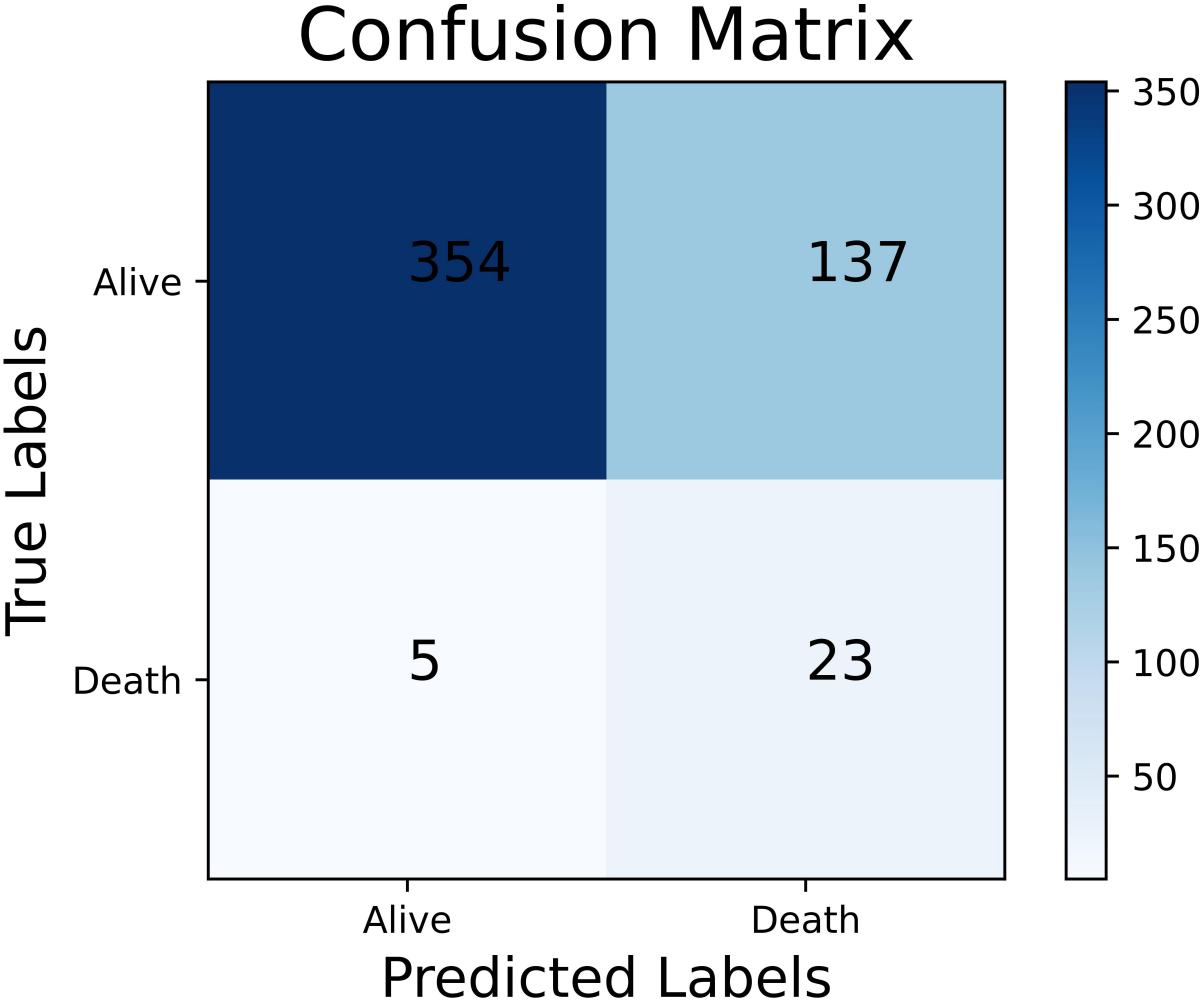
**The confusion matrix of the best performing female-specific model in women with STEMI**. STEMI, ST-elevation myocardial infarction.

**Fig. 9.**
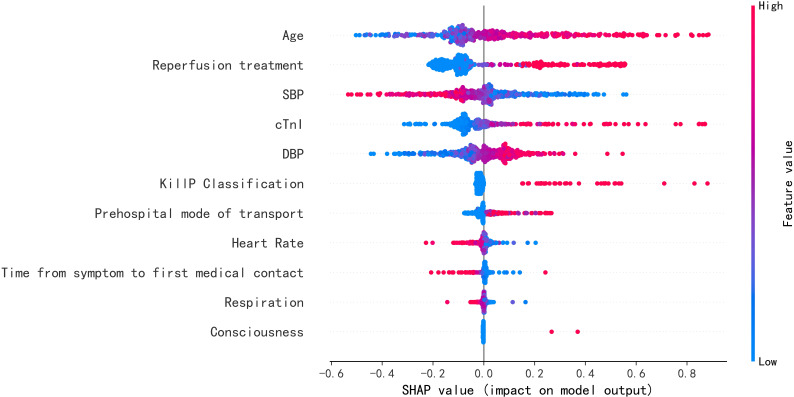
**SHAP values for mortality risk provided by the best performing female-specific model**. SBP, systolic blood pressure; cTnI, cardiac troponin I; DBP, diastolic blood pressure; SHAP, SHapley Additive exPlanations.

**Fig. 10.**
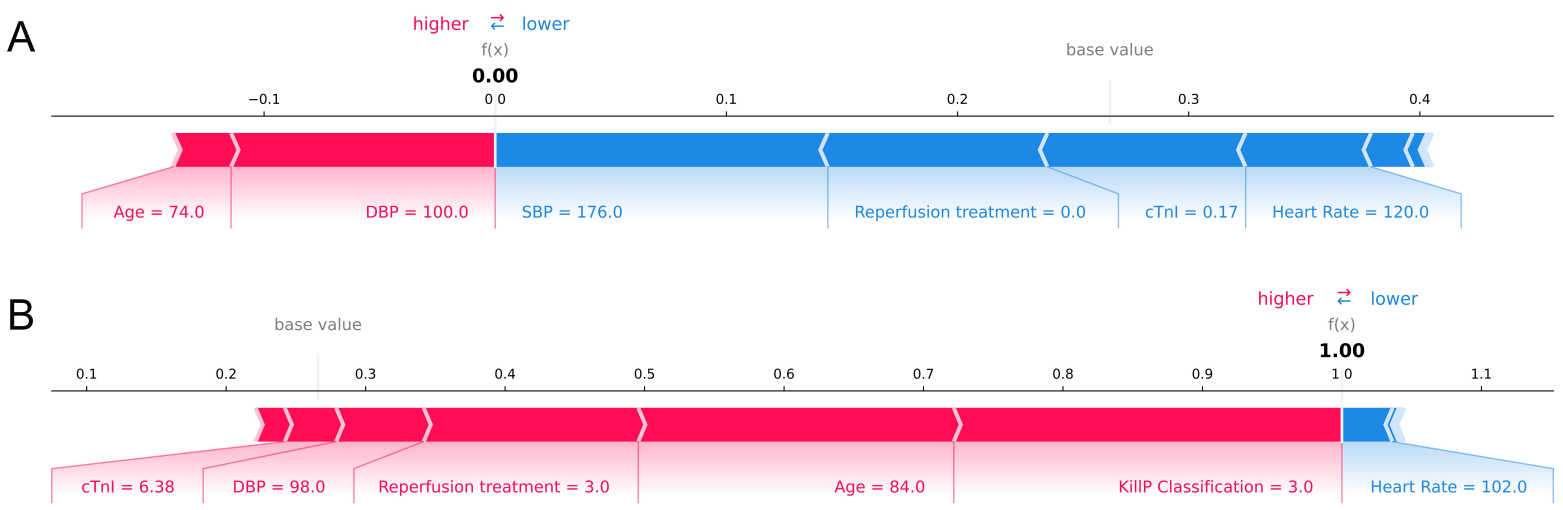
**The interpretation of model prediction results with the two samples**. (A) Survivor. (B) Non-survivor. SBP, systolic blood pressure; cTnI, cardiac troponin I; DBP, diastolic blood pressure.

The performance of different female-specific models is shown in Table 7 and the analysis of ROC curves is shown in Fig. 7. Similarly, the performance of models was significantly improved by ADASYN. The SVM combined with ADASYN achieved the best performance (G-mean: 76.74%; accuracy: 72.25%; sensitivity: 82.14%; specificity: 71.69%; and AUC: 79.26%), which significantly outperformed the best performing all-population-derived model in predicting in-hospital mortality in women with STEMI. Compared with the all-population-derived model, the accuracy and G-mean of the female-specific model increased by 34.64% (*p* = 0.029) and 9.07% (*p* = 0.027), respectively. The confusion matrix of the best performing female-specific model is shown in Fig. 8. Patients were correctly classified into the low-risk group (*n* = 354) and high-risk group (*n* = 23). The SHAP values are shown in Fig. 9. To further show the explainability of the model, two typical examples were provided as shown in Fig. 10: a 74-year-old woman who survived, and an 84-year-old woman who died. The arrows show the effect of each factor on the prediction. Specifically, the red arrows and blue arrows indicate that the factors increased and reduced the risk of death, respectively. The final SHAP value was provided by the combined influence of all factors and corresponded to the prediction score of the model. For the survivor, there was a low SHAP value (–0.2659) and prediction score (0.2210); for the non-survivor, there was a high SHAP value (0.7341) and prediction score (0.9825).

**Table 7.** **The performance of different female-specific models**.

Model	Accuracy	Specificity	Sensitivity	G-mean	AUC
DT	90.17%	93.89%	25.00%	48.45%	58.99%
DT_ADASYN	73.22%	73.32%	71.43%	72.37%	75.64%
RF	95.18%	100.00%	10.71%	32.73%	66.53%
RF_ADASYN	72.45%	72.30%	75.00%	73.64%	82.62%
SVM	93.06%	96.54%	32.14%	55.70%	56.71%
SVM_ADASYN	72.25%	71.69%	82.14%	76.74%	79.26%
XGBoost	94.99%	99.39%	17.86%	42.13%	70.05%
XGBoost_ADASYN	81.89%	82.89%	64.29%	73.00%	80.51%

DT, decision tree; RF, random forest; SVM, support vector machine; XGBoost, extreme gradient boosting; ADASYN, adaptive synthetic; AUC, area under the curve. G-mean is the geometric mean of sensitivity and specificity.

## 4. Discussion

STEMI is the leading cause of death among women worldwide [1, 2, 3, 4, 5, 6, 7, 8], which may be partly attributed to atypical symptoms and insufficient risk assessment. Therefore, in the present study, four commonly used ML algorithms were selected for the development of models to predict the in-hospital mortality in women with STEMI. Additionally, ADASYN was applied in order to improve the performance of the models [31]. The best performing female-specific model achieved an accuracy, sensitivity, specificity, G-mean, and AUC of 72.25%, 82.14%, 71.69%, 76.74% and 79.26%, respectively, leading to a more convenient and effective identification of high-risk patients at the first medical contact.

Consistent with previous studies [4, 41], our results demonstrated that women were more likely than men to have a delay between symptom and medical contact (121 min vs. 116 min, *p *= 0.0006), lower rates of reperfusion treatment (75.27% vs. 82.89%, *p <* 0.0001), and higher mortality (4.92% vs. 2.37%, *p <* 0.0001). The mechanisms behind these differences may be the following: (1) women with STEMI are more likely to present with multiple non-chest pain symptoms [19, 42, 43], which often results in an incorrect or delayed management; (2) competing responsibilities, as well as embarrassment or fear of disturbing others, lead women to be more likely to wait until symptoms subside rather than seek care [44]; and (3) because of lower socioeconomic status and lower perception for the risk of heart disease, women are less willing to opt for invasive coronary angiography [45, 46, 47]. As a result, physicians, patients, and relatives all tend to choose conservative treatments due to the lack of sex-specific guidelines [48]. Therefore, it is critical to optimize risk assessment and subsequent management, although the traditional risk-assessment tools are far from perfect. As machine learning blossoms, there are many successful applications of machine-learning models in the cardiovascular field. A machine-learning-based model called the PRAISE score was developed for predicting all-cause death, recurrent acute myocardial infarction, and major bleeding after acute coronary syndrome [49], but it was not designed for women. Recently, the GRACE 3.0 score, based on machine learning, was developed to reduce sex inequalities, but it was specifically developed for risk assessment of NSTE-ACS [7].

Although ML algorithms are accurate in capturing complex nonlinear relationships between clinical variables, when trained with imbalanced real-world medical data, the developed models are vulnerable to incorrectly predicting the minority class as the majority class [50], which leads the models to ignore high-risk patients. Therefore, an oversampling technology called ADASYN was applied to generate more samples from the minority class to alleviate the above problem. Compared with undersampling technology, which balances the Training Set by discarding the majority class samples, ADASYN can fully utilize precious medical data, resulting in a higher level of robustness [31]. Due to the “black-box” nature of ML models, the SHAP value was applied for explanation. The SHAP assesses the effect of each feature on results and presents it in an intuitive way [33], which can help doctors better understand how the model works, rather than blindly trusting the predictions.

The present study demonstrated that the female-specific models significantly outperformed the all-population-derived models in predicting in-hospital mortality in women with STEMI, and sex was considered to be an important predictor according to the feature importance scores (Fig. 4). However, women were not well represented in the study sample of the TIMI trial, where they accounted for only 24.7% [12], and in the study sample of the GRACE trial, where they accounted for 33.5% [11]. Additionally, our models can provide predictive results at the initial evaluation, resulting in an improvement in applicability. The 2017 ESC Guidelines recommend an aggressive treatment strategy for high-risk patients [34]. However, physicians, and women with STEMI, are more likely to choose conservative treatment (treatment-risk paradox), which can be inappropriate [51]. The proposed female-specific models can conveniently and effectively identify high-risk patients at the first medical contact, which can provide a basis for physicians to choose intensive treatment for high-risk patients, thereby improving treatment compliance.

This study has several limitations to be acknowledged. First, this is a single-center study. Therefore, the models should be validated in external centers to confirm their generalizability. Nonetheless, the risk prediction model proposed in this study still provides a convenient and effective method to predict in-hospital mortality in women with STEMI. Second, this is a retrospective study. Bias in patient enrollment and data collection is inevitable. However, the patients’ data were collected from a high-quality database, which reflected the real world. Third, the endpoint of this study included only in-hospital mortality, with no information on myocardial infarction, ischemic stroke, or heart failure; information on longitudinal follow-up was not obtained. Thus, further long-term follow-up studies are needed to obtain more detailed and comprehensive information in order to develop more clinically instructive models. Finally, some important predictors were not included in this study, such as creatinine level, myocardial injury biomarkers, and sex-specific risk factors, which attenuated the performance of models and made the comparison to other risk scores impossible. Conversely, our models can be used conveniently and effectively in pre-hospital or emergency departments. In addition, the symptoms of MI are usually atypical in elderly patients and in patients with diabetes, which makes these patients less willing to seek medical service. Therefore there are many papers and literature works that focus on diabetes and the elderly as distinct groups [52, 53, 54, 55]. Accordingly, future studies should focus on applying machine learning to improve the prognosis of these patients.

## 5. Conclusions

In this study, four commonly used ML models (DT, RF, and SVM) were developed to predict in-hospital mortality in women with STEMI. The predictors could be obtained at initial evaluation. Additionally, ADASYN was applied to assess and mitigate the effects of class imbalance, thereby improving model performance. By capturing the non-linear association of predictors, the proposed female-specific model could conveniently and effectively identify high-risk female patients at the first medical contact. Therefore, the integration of our female-specific model into daily clinical practice may improve the prognosis of women with STEMI who often suffer from an incorrect or delayed diagnosis.

## Data Availability

The datasets used and/or analyzed during the current study are available from the corresponding author on reasonable request.

## References

[1] Chen L, Hu Z, Wang X, Song Y, Chen Z, Zhang L (2022). Age at Menarche and Menopause, Reproductive Lifespan, and Risk of Cardiovascular Events Among Chinese Postmenopausal Women: Results From a Large National Representative Cohort Study. *Frontiers in Cardiovascular Medicine*.

[2] Chiesa M, Piacentini L, Bono E, Milazzo V, Campodonico J, Marenzi G (2020). Whole blood transcriptome profile at hospital admission discriminates between patients with ST-segment elevation and non-ST-segment elevation acute myocardial infarction. *Scientific Reports*.

[3] Ma H, Guo L (2017). Simultaneous acute occlusion of right and anterior descending coronary arteries in acute myocardial infarction in a young man. *Heart & Mind*.

[4] Pagidipati NJ, Peterson ED (2016). Acute coronary syndromes in women and men. *Nature Reviews Cardiology*.

[5] Ardissino M, Nelson AJ, Maglietta G, Malagoli Tagliazucchi G, Disisto C, Celli P (2022). Sex-Related Differences in Long-Term Outcomes After Early-Onset Myocardial Infarction. *Frontiers in Cardiovascular Medicine*.

[6] Shabbir A, Rathod KS, Khambata RS, Ahluwalia A (2021). Sex Differences in the Inflammatory Response: Pharmacological Opportunities for Therapeutics for Coronary Artery Disease. *Annual Review of Pharmacology and Toxicology*.

[7] Wenzl FA, Kraler S, Ambler G, Weston C, Herzog SA, Räber L (2022). Sex-specific evaluation and redevelopment of the GRACE score in non-ST-segment elevation acute coronary syndromes in populations from the UK and Switzerland: a fmultinational analysis with external cohort validation. *Lancet*.

[8] Haider A, Bengs S, Luu J, Osto E, Siller-Matula JM, Muka T (2020). Sex and gender in cardiovascular medicine: presentation and outcomes of acute coronary syndrome. *European Heart Journal*.

[9] Zhao D, Smith SC (2021). Quality of care for patients with acute coronary syndrome. *Cardiology Plus*.

[10] Xiang DC, Jin YZ, Fang WY, Su X, Yu B, Wang Y (2021). The national chest pain centers program: Monitoring and improving quality of care for patients with acute chest pain in China. *Cardiology Plus*.

[11] Granger CB, Goldberg RJ, Dabbous O, Pieper KS, Eagle KA, Cannon CP (2003). Predictors of hospital mortality in the global registry of acute coronary events. *Archives of Internal Medicine*.

[12] Morrow DA, Antman EM, Charlesworth A, Cairns R, Murphy SA, de Lemos JA (2000). TIMI risk score for ST-elevation myocardial infarction: A convenient, bedside, clinical score for risk assessment at presentation: An intravenous nPA for treatment of infarcting myocardium early II trial substudy. *Circulation*.

[13] Eagle KA, Lim MJ, Dabbous OH, Pieper KS, Goldberg RJ, Van de Werf F (2004). A validated prediction model for all forms of acute coronary syndrome: estimating the risk of 6-month postdischarge death in an international registry. *The Journal of the American Medical Association*.

[14] Fox KAA, Fitzgerald G, Puymirat E, Huang W, Carruthers K, Simon T (2014). Should patients with acute coronary disease be stratified for management according to their risk? Derivation, external validation and outcomes using the updated GRACE risk score. *BMJ Open*.

[15] Zhao M, Vaartjes I, Graham I, Grobbee D, Spiering W, Klipstein-Grobusch K (2017). Sex differences in risk factor management of coronary heart disease across three regions. *Heart*.

[16] Banco D, Chang J, Talmor N, Wadhera P, Mukhopadhyay A, Lu X (2022). Sex and Race Differences in the Evaluation and Treatment of Young Adults Presenting to the Emergency Department With Chest Pain. *Journal of the American Heart Association*.

[17] Minissian MB, Mehta PK, Hayes SN, Park K, Wei J, Bairey Merz CN (2022). Ischemic Heart Disease in Young Women: JACC Review Topic of the Week. *Journal of the American College of Cardiology*.

[18] Gabani R, Spione F, Arevalos V, Grima Sopesens N, Ortega-Paz L, Gomez-Lara J (2022). Sex Differences in 10-Year Outcomes Following STEMI: A Subanalysis From the EXAMINATION-EXTEND Trial. *JACC: Cardiovascular Interventions*.

[19] Tran VH, Mehawej J, Abboud DM, Tisminetzky M, Hariri E, Filippaios A (2022). Age and Sex Differences and Temporal Trends in the Use of Invasive and Noninvasive Procedures in Patients Hospitalized With Acute Myocardial Infarction. *Journal of the American Heart Association*.

[20] Sun GW, Shook TL, Kay GL (1996). Inappropriate use of bivariable analysis to screen risk factors for use in multivariable analysis. *Journal of Clinical Epidemiology*.

[21] Breiman L (2001). Statistical modeling: The two cultures (with comments and a rejoinder by the author). *Statistical Science*.

[22] Bagley SC, White H, Golomb BA (2001). Logistic regression in the medical literature: standards for use and reporting, with particular attention to one medical domain. *Journal of Clinical Epidemiology*.

[23] Hand DJ (1998). Data mining: statistics and more. *The American Statistician*.

[24] Zhao J, Zhao P, Li C, Hou Y (2021). Optimized Machine Learning Models to Predict In-Hospital Mortality for Patients with ST-Segment Elevation Myocardial Infarction. *Therapeutics and Clinical Risk Management*.

[25] Wang K, Tian J, Zheng C, Yang H, Ren J, Liu Y (2021). Interpretable prediction of 3-year all-cause mortality in patients with heart failure caused by coronary heart disease based on machine learning and SHAP. *Computers in Biology and Medicine*.

[26] Motwani M, Dey D, Berman DS, Germano G, Achenbach S, Al-Mallah MH (2017). Machine learning for prediction of all-cause mortality in patients with suspected coronary artery disease: a 5-year multicentre prospective registry analysis. *European Heart Journal*.

[27] Angraal S, Mortazavi BJ, Gupta A, Khera R, Ahmad T, Desai NR (2020). Machine Learning Prediction of Mortality and Hospitalization in Heart Failure With Preserved Ejection Fraction. *JACC: Heart Failure*.

[28] Abbasi MU, Rashad A, Srivastava G, Tariq M (2022). Multiple contaminant biosignal quality analysis for electrocardiography. *Biomedical Signal Processing and Control*.

[29] Lin WC, Tsai CF, Hu YH, Jhang JS (2017). Clustering-based undersampling in class-imbalanced data. *Information Sciences*.

[30] Mohammed R, Rawashdeh J, Abdullah M (2020). Machine learning with oversampling and undersampling techniques: overview study and experimental results. 2020 11th international conference on information and communication systems (ICICS). *IEEE*.

[31] He H, Bai Y, Garcia EA, Li S (2008). ADASYN: Adaptive synthetic sampling approach for imbalanced learning. 2008 IEEE international joint conference on neural networks (IEEE world congress on computational intelligence). *IEEE*.

[32] Castiglioni I, Rundo L, Codari M, Di Leo G, Salvatore C, Interlenghi M (2021). AI applications to medical images: From machine learning to deep learning. *Physica Medica*.

[33] Lundberg SM, Lee SI (2017). A unified approach to interpreting model predictions. *Advances in Neural Information Processing Systems*.

[34] Ibanez B, James S, Agewall S, Antunes MJ, Bucciarelli-Ducci C, Bueno H (2018). 2017 ESC Guidelines for the management of acute myocardial infarction in patients presenting with ST-segment elevation: The Task Force for the management of acute myocardial infarction in patients presenting with ST-segment elevation of the European Society of Cardiology (ESC). *European Heart Journal*.

[35] Tokodi M, Schwertner WR, Kovács A, Tősér Z, Staub L, Sárkány A (2020). Machine learning-based mortality prediction of patients undergoing cardiac resynchronization therapy: the SEMMELWEIS-CRT score. *European Heart Journal*.

[36] Dietterich T (1998). Approximate Statistical Tests for Comparing Supervised Classification Learning Algorithms. *Neural Computation*.

[37] Štrumbelj E, Kononenko I (2014). Explaining prediction models and individual predictions with feature contributions. *Knowledge and information systems*.

[38] Lundberg SM, Nair B, Vavilala MS, Horibe M, Eisses MJ, Adams T (2018). Explainable machine-learning predictions for the prevention of hypoxaemia during surgery. *Nature Biomedical Engineering*.

[39] Athanasiou M, Sfrintzeri K, Zarkogianni K, Thanopoulou AC, Nikita KS (2020). An explainable XGBoost–based approach towards assessing the risk of cardiovascular disease in patients with Type 2 Diabetes Mellitus. 2020 IEEE 20th International Conference on Bioinformatics and Bioengineering (BIBE). *IEEE*.

[40] Jiang Z, Bo L, Xu Z, Song Y, Wang J, Wen P (2021). An explainable machine learning algorithm for risk factor analysis of in-hospital mortality in sepsis survivors with ICU readmission. *Computer Methods and Programs in Biomedicine*.

[41] Chen SQ, Liu J, Zhou Y, Huang ZD, Xie Y, Huang HZ (2022). Sex Differences in Characteristics, Treatments, and In-hospital Outcomes of Patients Undergoing Coronary Angiography or Intervention. *Frontiers in Cardiovascular Medicine*.

[42] van Oosterhout REM, de Boer AR, Maas AHEM, Rutten FH, Bots ML, Peters SAE (2020). Sex Differences in Symptom Presentation in Acute Coronary Syndromes: A Systematic Review and Meta-analysis. *Journal of the American Heart Association*.

[43] Walli-Attaei M, Joseph P, Rosengren A, Chow CK, Rangarajan S, Lear SA (2020). Variations between women and men in risk factors, treatments, cardiovascular disease incidence, and death in 27 high-income, middle-income, and low-income countries (PURE): a prospective cohort study. *Lancet*.

[44] Lichtman JH, Leifheit EC, Safdar B, Bao H, Krumholz HM, Lorenze NP (2018). Sex Differences in the Presentation and Perception of Symptoms Among Young Patients With Myocardial Infarction: Evidence from the VIRGO Study (Variation in Recovery: Role of Gender on Outcomes of Young AMI Patients). *Circulation*.

[45] D’Onofrio G, Safdar B, Lichtman JH, Strait KM, Dreyer RP, Geda M (2015). Sex differences in reperfusion in young patients with ST-segment-elevation myocardial infarction: results from the VIRGO study. *Circulation*.

[46] DeFilippis EM, Collins BL, Singh A, Biery DW, Fatima A, Qamar A (2020). Women who experience a myocardial infarction at a young age have worse outcomes compared with men: the Mass General Brigham YOUNG-MI registry. *European Heart Journal*.

[47] Stehli J, Dinh D, Dagan M, Duffy SJ, Brennan A, Smith K (2021). Sex Differences in Prehospital Delays in Patients With ST-Segment-Elevation Myocardial Infarction Undergoing Percutaneous Coronary Intervention. *Journal of the American Heart Association*.

[48] Daugherty SL, Blair IV, Havranek EP, Furniss A, Dickinson LM, Karimkhani E (2017). Implicit Gender Bias and the Use of Cardiovascular Tests Among Cardiologists. *Journal of the American Heart Association*.

[49] D’Ascenzo F, De Filippo O, Gallone G, Mittone G, Deriu MA, Iannaccone M (2021). Machine learning-based prediction of adverse events following an acute coronary syndrome (PRAISE): a modelling study of pooled datasets. *Lancet*.

[50] Guo HX, Li YJ, Shang J, Gu MY, Huang YY, Bing G (2017). Learning from class-imbalanced data: Review of methods and applications. *Expert Systems with Applications*.

[51] Shah T, Haimi I, Yang Y, Gaston S, Taoutel R, Mehta S (2021). Meta-Analysis of Gender Disparities in In-hospital Care and Outcomes in Patients with ST-Segment Elevation Myocardial Infarction. *The American Journal of Cardiology*.

[52] Díez-Villanueva P, Jiménez-Méndez C, Bonanad C, Ortiz-Cortés C, Barge-Caballero E, Goirigolzarri J (2022). Sex differences in the impact of frailty in elderly outpatients with heart failure. *Frontiers in Cardiovascular Medicine*.

[53] Sanz-Girgas E, Peiró ÓM, Bonet G, Rodríguez-López J, Scardino C, Ferrero-Guillem M (2021). A simple combination of biomarkers for risk stratification in octogenarians with acute myocardial infarction. *Reviews in Cardiovascular Medicine*.

[54] Lin Y, Liu F, Gong S, Liao B, Liu H, Yuan J (2021). Validity of SOFA score as a prognostic tool for critically ill elderly patients with acute infective endocarditis. *Reviews in Cardiovascular Medicine*.

[55] Jiang H, Feng J, Feng C, Ren P, Ren K, Jin Y (2022). Validation and Comparison of PROMISE and CONFIRM Model to Predict High-Risk Coronary Artery Disease in Symptomatic and Diabetes Mellitus Patients. *Reviews in Cardiovascular Medicine*.

